# The witchweed *Striga gesnerioides* and the cultivated cowpea: A geographical and historical analysis of their West African distribution points to the prevalence of agro-ecological factors and the parasite’s multilocal evolution potential

**DOI:** 10.1371/journal.pone.0254803

**Published:** 2021-08-04

**Authors:** Abou-Soufianou Sadda, Geo Coppens d’Eeckenbrugge, Abdoul-Aziz Saidou, Abdoulaye Diouf, Nouhou Salifou Jangorzo, Hassane Bil-Assanou Issoufou, Oumarou Malam-Issa

**Affiliations:** 1 UMR DAP, Université Dan Dicko Dankoulodo de Maradi, Maradi, Niger; 2 IRD, UMR IEES-Paris, SU/IRD/CNRS/INRA/UPEC/Univ. Paris Diderot, Centre IRD de France Nord, Bondy Cedex, France; 3 CIRAD, UMR AGAP, Montpellier, France; 4 AGAP, Univ. Montpellier, CIRAD, INRAE, Institut Agro, Montpellier, France; 5 UMR ECODYV, Université Dan Dicko Dankoulodo de Maradi, ADS Maradi, Niger; University of Molise, Isernia, ITALY

## Abstract

The increasing severity of *Striga gesnerioides* attacks on cowpea across West Africa has been related to its prolificity, seed mobility and longevity, and adaptation to aridity, in a context of agricultural intensification. To understand this fast extension, we analyzed (1) the distributions of the crop and the witchweed with ecological niche modeling and multivariate climate analysis, and (2) the chronological information available from collections and the literature. The ecoclimatic envelope of *S*. *gesnerioides* attacks on cowpea is the same as on wild hosts. Consistently, the modeled distribution of cowpea infestations is closely similar to the simple superposition of the parasite model (involving all hosts) and the crop model. *Striga gesnerioides* infestations are restricted to the driest component of the cultivated cowpea ecoclimatic niche, corresponding to the Sahelian and Sudano-Sahelian belts and the Dahomey gap. Thus, the parasite distribution, determined by its own requirements, does not constrain cowpea cultivation under Guinean climates. The spatial and temporal distributions of *S*. *gesnerioides* field infestations are consistent with an earlier impact on cowpea production in eastern West Africa, related itself to a similar trend in cowpea cultivation intensification from Niger, Nigeria and Benin to Burkina Faso and Ghana. Mali and Senegal were affected later, and literature reports of Senegalese strains of *S*. *gesnerioides* from the wild developing virulence on cowpea offer a model for the diffusion of witchweed parasitism by multilocal evolution, through host-driven selection, instead of epidemic diffusion. *A contrario*, in Côte d’Ivoire, cowpea is much less widespread, so the parasite has remained confined to the wild compartment. Thus, both historical and ecogeographic analyses refute the vision of *S*. *gesnerioides* as an invader. Instead, they point to the increasing importance and intensification of the crop, and the consequent loss of biodiversity, as the main drivers of the extension and diversification of its crop-specific strains.

## Introduction

Cowpea [*Vigna unguiculata* (L.) Walp.] is one of the most widely grown legume crops throughout the tropics and subtropics of Africa, East and Southeast Asia, Latin America, parts of southern Europe, as well as in the southern United States and in Oceania, with a 2017 world production above 7.4 million tons [1]. Its production is by far most important in Africa (7.1 Mt), particularly in West Africa, with Nigeria (3.4 Mt) and Niger (1.96 Mt) as the main producing countries. It has more than tripled since the mid-1980s in Cameroon, Nigeria, Niger, Burkina Faso, Mali, and Senegal, mostly based on an extension of cultivated areas [2]. Cowpea has a greater ability to withstand the frequent droughts of the Sahelian and Sudanian zones than any other major crop [3]. Consumed for its grains, green pods or leaves, it provides an inexpensive source of plant protein and mineral elements [4], particularly micronutrients such as iron and zinc [5] that can improve the nutritional status of resource-poor subsistence farmers [6]. Furthermore, cowpea contribution to soil fertility, through soil covering and the fixation of atmospheric nitrogen, is particularly important in smallholder farming systems where limited or no fertilizers are used [7].

However, in West and Central Africa, cowpea faces severe abiotic and biotic constraints. Abiotic stresses mostly include severe drought [8, 9] and salinity [10, 11]. Biotic pests include bacterial, fungal and viral diseases [12], insects [13], nematodes [14], herbivores [15], and particularly the parasitic weed *Striga gesnerioides* (Willd.) Vatke (Scrophulariaceae), an obligate root-parasitic flowering plant [16, 17]. The latter is the most widely distributed *Striga* species across Africa with extensions to Arabia and Asia between 33°10’N and 32°15’S [18], also causing problems on indigo (*Indigofera tinctoria* L.) [19] in Senegal, tobacco (*Nicotiana tabacum* L.) in Southern Africa and sweet potato (*Ipomoea batatas* (L.) Lam) in East Africa [20]; in pot tests, it is also known to attack sunflower (*Helianthus annuus* L.) [19]. Other dicotylodenous hosts for *S*. *gesnerioides* include wild members of Fabaceae (genera *Alysicarpus*, *Indigofera*, *Tephrosia*, *Crotalaria*, *Cassia*, *Dalbergia*, and *Zornia*) and Convolvulaceae (genera *Ipomoea*, *Jaquemontia*, *Merremia*), Acanthaceae (genera *Lepidagathis* and *Dysophylla*, species of genus *Euphorbia*, and *Richardia scabra* L. (Rubiaceae) [21–24]. According to most authors, the parasitism relationship with its different host species is highly specific [16, 19, 25] regarding the mode of germination, fixation and development. Similarly, at intraspecific levels, the resistances against different races of the parasite vary among cowpea cultivars and landraces [26, 27]. However, while confirming morphological differences between *S*. *gesnerioides* strains found on natural vegetation (genera *Ipomoea* and *Indigofera*) and those found on cultivated cowpea, Wade [24] showed that seeds from both sources were able to germinate and infect both types of plants, accomplishing the whole parasite cycle.

As stated by Ejeta [28] “the *Striga* problem in Africa is exasperated by its exquisite adaptation to the climatic conditions of the semi-arid tropics, its high fecundity and longevity of its seed reserves in tropical soils.” Indeed, with an estimated 100 million ha of African savannahs infested annually [28], *Striga* species, particularly *S*. *gesnerioides* for legumes, as well as *S*. *hermonthica* (Del.) Benth. and *S*. *asiatica* (L.) Kuntze for cereals, have greater impact on human welfare and sustainable food production in tropical and subtropical areas than any other parasitic angiosperm because they interfere with subsistence crops in semi-arid areas that are marginal for agriculture. *Striga* has become a significant problem from the late 1970’s [29], in relation to population pressure and the intensification of land use. *Striga* populations have increased with monocropping, abandonment or reduction of fallows, soil fertility degradation and the introduction of exogenous germplasm that had not evolved under *Striga* pressure [16, 17, 20, 25, 30, 31]. In Burkina Faso, Aggarwal and Ouedraogo [32] and Muleba et al. [17] reported average yield losses of 30% in cowpea cultivars susceptible to *S*. *gesnerioides*. More severe yield losses (28 to 59%) have been reported for susceptible genotypes from an experiment in northern Ghana [33]. Severe infestations can even cause total crop failure [16].

A single capsule of *S*. *gesnerioides* contains 400–500 seeds (measuring 0.20–0.35 mm [16] that may remain viable for 14 years in the soil, as demonstrated for *S*. *asiatica* [34]. They can disseminate easily, through water, wind, animals, farming tools and seed markets, however only the latter appears to be effective for long distance dispersal [25, 31].

Considering that “the least expensive *Striga* management strategies are those devised on the basis of prediction, prevention of invasion, early detection, and containment,” Mohamed et al. [22] developed global ecological niche models (ENM) of potential distribution for ten witchweeds, including five *Striga* species. Such models assess the relationships between species records and the environmental characteristics of the corresponding observation sites, and then predict relative occurrence rates as a function of the environmental predictors for each location on a map. Based on occurrences from Central and Southern Africa, Mohamed et al. [22] found that *S*. *asiatica*, *S*. *hermonthica*, and *S*. *gesnerioides* present great invasive potential, which they attributed to their ability to adapt to different habitats and agroecosystems by developing host-specific strains, each capable of attacking a narrow host range. However, in such a general study, a possible effect of host-parasite interactions on *Striga* distribution was not tested.

Cotter et al. [35] assessed *S*. *hermonthica* African distribution using the Worldclim database, and projecting it on two models predicting the 2020 climate. More recently, Bellis et al. [36] produced an African distribution model for the same species, based on bioclimatic, topographic and soil variables. They reported (i) that annual rainfall and total soil nitrogen were the most informative variables to predict *S*. *hermonthica* occurrence; and (ii) a high degree of overlap between models generated using either all *S*. *hermonthica* records or a subset of occurrences that were observed specifically in sorghum fields. This overlap suggests that the distribution of the parasite may not be related to host diversity, contradicting the hypothesis of Mohamed et al. [22].

Given the very high social and economic importance of cowpea and the increasing severity of *S*. *gesnerioides* infestation in West Africa, the present study focuses on the ecological drivers of the interaction between the parasite and its main host in this region. It combines the use of Maxent ENM software [37] with multivariate analyses on bioclimatic covariates to characterize the environmental envelopes of both species. Furthermore, it uses chronological information from collected specimens and the literature to compare past and present distributions of *S*. *gesnerioides*.

## Materials and methods

**Fig 1:**
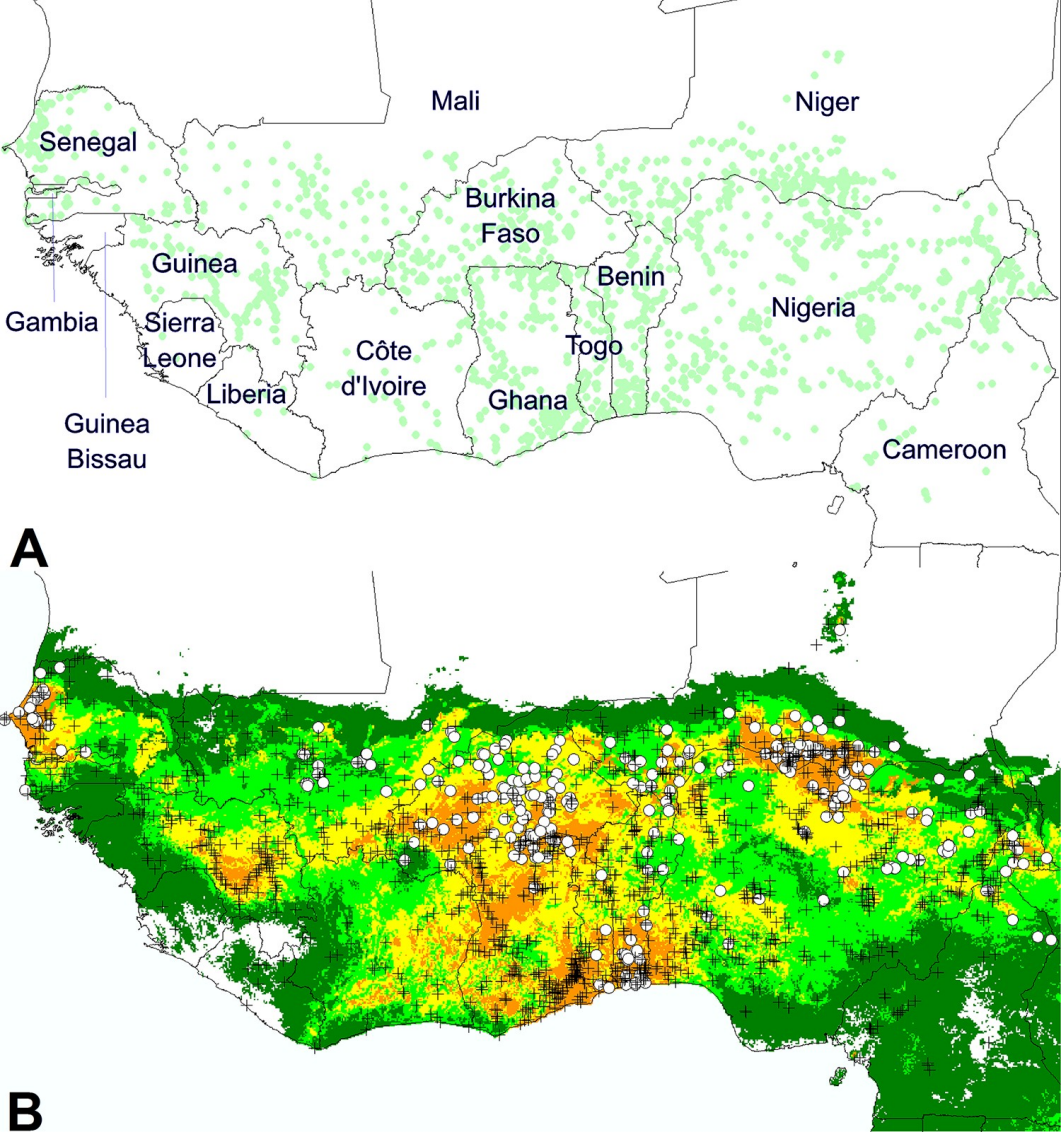
Geographic distribution of cultivated cowpea in West Africa. **A.** Region of study and global distribution of the 1747 datapoints of occurrence. **B.** Ecoclimatic model of cultivated cowpea distribution in West Africa. Cowpea datapoints are represented by black crosses, except for cases of cowpea infected by *S*.*gesnerioides*, represented by white circles. Background color indicates climate suitability: unsuitable (no color); marginal (dark green); favorable (light green; above 10 percentile training presence); very good (yellow; above 33 percentile training presence); excellent (orange; above 67 percentile training presence).

The study focused on West African countries between the Sahelian belt and the Gulf of Guinea. From east to west, this area includes Cameroon, Niger, Nigeria, Benin and Togo, Burkina Faso, Mali and Ghana, Côte d’Ivoire, Liberia, Sierra Leone, Guinea, Guinea Bissau, Gambia and Senegal (Fig 1A). Three inter-related georeferenced datasets were developed: occurrences of cultivated cowpea, occurrences of cultivated cowpea infestation by *S*. *gesnerioides*, and occurrences of *S*. *gesnerioides* on all its hosts in the region. As wild or feral cowpeas, either annual or perennial, differ from cultivated cowpea in their phenology and adaptation to aridity and seasonality [38], occurrences of spontaneous forms were excluded from the cowpea dataset. The study of their distribution should integrate other components of their niche, mainly survival in the dry season, competition and predation.

The collated and validated information was first used for mapping the realized geographic distribution of both the crop and the witchweed. For the latter, the information associated with the occurrence points was filtered (i) by date of the collections/observations and (ii) by host species. Second, it was used to develop ecoclimatic models for (i) the crop, (ii) the crop infestation, and (iii) the parasite on all its hosts. The crop infestation model was then compared to the superposition of both the crop and the parasite models, to test the dependence of the parasite niche on that of the crop. Every modeling exercise was complemented by a characterization of the bioclimatic envelopes (or spaces) corresponding to each distribution model, based on a principal component analysis (PCA) of the main contributing variables.

### Development of species occurrence datasets

Geographical and ecological information about cowpea and *S*. *gesnerioides* were obtained from (i) field campaigns performed within the framework of the CowpeaSquare project in Niger (443 datapoints on both species), of which this study was a part; (ii) the CoEx project (regional study including surveys on cowpea in Niger, Mali, Senegal and Burkina Faso; 144 datapoints on cowpea); (iii) a study on cowpea biodiversity (surveys, collection, characterization) at CERAAS/ISRA (Centre d’Étude Régional pour l’Amélioration de l’Adaptation à la Sécheresse/Institut Sénégalais de Recherche Agronomique) in Senegal (37 datapoints on cowpea), (iv) from public repository online presence records included in the Global Biodiversity Information Facility (GBIF, https://www.gbif.org) and Tropicos (http://www.tropicos.org), with 27,153 datapoints on cowpea and 719 on *S*. *gesnerioides*; (v) the herbarium collections of the Museum National d’Histoire Naturelle, Paris (MNHN) and the Herbier National du Bénin (respectively 242 and 12 datapoints on *S*. *gesnerioides*); and (vi) from relevant literature (210 and 224 datapoints respectively on cowpea and *S*. *gesnerioides*). This information is synthetized in S1A Table.

Coordinates were checked for congruence with administrative boundaries with Diva-GIS software version 7.5.0. Incomplete datalines or fuzzy occurrences with vague descriptions were discarded, as well as redundant data; the conserved occurrences were proofread against associated geographic information, using systematically the Geonames gazetteer (http://www.geonames.org/) and Google Earth. Two levels of data quality were distinguished: high precision georeferencing that includes GPS coordinates or matches exactly associated locality information; and occurrences with medium precision, i.e., with coordinates verifiable to the village location. Among the GBIF data, 26,038 and 638 datalines were thus eliminated for the crop and the parasite respectively (S1B Table). In those areas concentrating a significant proportion of occurrences (e.g. in Niger), the density of observations was further reduced, based on criteria of more uniform observation density across the whole studied region, geolocalization precision and quality of information associated with each particular record, with the aim of avoiding possible sampling biases among countries. The final datasets included 1747 and 425 datapoints for cowpea and *S*. *gesnerioides* respectively (S1C Table).

### Environmental layers

For each occurrence record, 19 bioclimatic layers (averaged over the 1970–2000 time range), with a spatial resolution of 2.5 arc-min (4.63 km at the equator), were downloaded from the online World Climate website (https://www.worldclim.org/data/worldclim21.html; [39]). These variables are: 1) annual mean temperature; 2) mean diurnal range (mean of monthly (max temp–min temp); 3) isothermality (Bio2/Bio7); 4) temperature seasonality; 5) maximal temperature of warmest month; 6) minimal temperature of coldest month; 7) temperature annual range; 8) mean temperature of wettest quarter; 9) mean temperature of driest quarter; 10) mean temperature of warmest quarter; 11) mean temperature of coldest quarter; 12) annual precipitation; 13) precipitation of wettest month; 14) precipitation of driest month; 15) precipitation seasonality; 16) precipitation of wettest quarter; 17) precipitation of driest quarter; 18) precipitation of warmest quarter; and 19) precipitation of coldest quarter.

Soil covariates, such as soil total nitrogen, were not included, because of the limitations of available gridded information, for three reasons. First, this information has been developed through machine learning modeling where some of the involved covariates are obtained from remote sensing (e.g. vegetation indices, land cover classes) and other gridded databases (including bioclimatic variables) [40]. Thus, while climate is an important driver of soil formation, there is a strong risk that soil covariate prediction itself is too dependent on the bioclimatic covariates used in our modeling exercise, or on the vegetation cover, obviously related to its expected output. For example, the similarity observed between the modeled total nitrogen grid and the distribution of forests, or the strong correlation between total N and annual rainfall (r = 0.84 in our region of study), are highly problematic for the interpretation of a model combining climate and soil data. Second, despite considerable improvements, arid and semi-arid regions are still largely under-represented in the training dataset for soil modeling [40], which is particularly problematic for this study. Third, the precision in the geolocalization of collected plants is too often inconsistent with the high resolution of soil gridded models (250 m), while the insufficient spatial resolution of the soil maps and/or georeferencing errors reduce their predictive ability [41].

### Ecoclimatic niche modeling

We used the Maxent 3.4.1 [42] presence-only modeling software because of its excellent performance relatively to most other methods [37]. Maxent was run with the default variable responses settings, and a logistic output format that results in a map of habitat suitability of the species ranging from 0 to 1 per grid cell. Classically, a logistic threshold value equivalent to the 10 percentile training presence was retained to separate climatically favorable areas from marginally fit areas. Among marginally fit areas, those under a threshold value corresponding to 0.5% training presence were discarded. Among climatically favorable areas, the values corresponding to percentiles 33 and 67 were used to distinguish ‘favorable conditions’, ‘very good conditions’, and ‘excellent conditions.’ Thus, the different distribution models could be mapped in a standardized manner.

### Characterization of ecoclimatic envelopes

The relative contributions of the 19 bioclimatic predictors to the distribution model were assessed with the jackknife test presented in the Maxent outcome. The PCA was performed on the most informative bioclimatic variables to characterize and compare the climatic envelopes of the two species, discarding those variables whose contribution appeared marginal and non-specific. Only principal components with an eigenvalue over 1 were retained and a normalized varimax rotation was applied to maximize the sum of the variances of the squared loadings, simplifying the interpretation of the results [43]. The visualization of individual observations in the principal plane allowed comparing their relative situations within the species’ ecoclimatic envelope and the relative contributions of data from different countries of the region.

## Results and discussion

### Geographic distribution of cultivated cowpea: The data and the model

The distribution of the 1747 retained datapoints (Fig 1A) represents the geographic range of cultivated cowpea at the West African scale. The datapoint density appears heterogeneous across countries of interest. It is very high from northern Cameroon to Ghana and Burkina Faso, passing through Nigeria, southern and western Niger, Benin, and Togo. More to the west, datapoints appear less dense, yet relatively well distributed between southern Mali, northwestern Côte d’Ivoire, Guinea, and western Senegal, whereas observations are very sparse in Gambia, Sierra Leone, and Liberia. In the eastern half of Senegal and the western half of southern Mali, the uniform and relatively low density is linked to the source of data, mostly the CoEx project, which followed a particular sampling strategy. Otherwise, this relative east-west contrast probably reflects the economic importance of the crop among West African countries, with the relative exception of Nigeria, the main producer country, where the datapoint density is lower than for the second and third producers, Niger and Burkina Faso. More to the east, datapoint density in Mali may not represent its substantial contribution to world production [1].

Fig 1B presents the modeled distribution obtained with the Maxent software for cultivated cowpea in West Africa. Very good to excellent climatic conditions dominate in the northeast zone (Niger and northern Nigeria) and in the southern central zone, in and around the Dahomey gap (southern Nigeria, Benin, Togo, Ghana, southern Burkina Faso, and most of Côte d’Ivoire). In Côte d’Ivoire, these highly favorable conditions do not appear to match extensive cowpea cultivation. To the west, we find a highly favorable area in Guinea, and another one in Senegal, where the slight contrast between climatic adaptation and relative datapoint dispersal can be explained by observation methodology and/or a recent extension of the crop. In fact, cowpea has much increased in importance as a food crop in the northern peanut basin of Senegal after the repeated droughts of the late 20^th^ century, partly substituting more traditional staples, due to its ability to produce food in infertile soils under these conditions [24].

To validate this model, we have confronted it to the expert information presented in the report of the Organization for Economic Co-operation and Development (OECD) [3], observing only partial correspondence. In fact, cowpea cultivation areas correspond very well to our Maxent model in the semi-arid Sahelian to Sudano-Sahelian zone and the northern Guinean savannah zone. However, the two maps differ significantly for the southern and western Guinean savannah and forest zones of West Africa. To the west, the OECD map also shows no significant cowpea cultivation not only from Guinea Bissau, through Sierra Leone, to Liberia, but also in the highly favorable region of northeastern Guinea, where our occurrence data document frequent cowpea cultivation in the latter region of Guinea. Along the shores of the Gulf of Guinea, the OECD map only shows little cowpea cultivation in the south of Côte d’Ivoire, Ghana, Togo, and Benin. For Nigeria, it appears limited to the north of the country, and the OECD distribution does not include most of the light green areas reported for this country in Fig 1B. In contrast, our occurrence data indicate limited cultivation in Côte d’Ivoire, but frequent cultivation in Ghana, Togo, Benin and Nigeria, even in the south, all regions where, according to the Maxent model, cowpea benefits from good to excellent climatic conditions.

### Geographic distribution of *Striga gesnerioides* infestations on cultivated cowpea

From the 425 records of *S*. *gesnerioides*, 275 correspond to infestations on cultivated cowpea. As shown in Fig 1B, most of them were from regions with highly suitable climates for cowpea cultivation. This is particularly true in the center and to the east of the study area (i.e., from northern Cameroon to Burkina Faso and Togo). In the south, along the Gulf of Guinea, records of *S*. *gesnerioides* infestations were limited to the Dahomey gap, with presence in Benin and Togo. We have found no infestation records from southern Ghana to Guinea Bissau. In the case of Côte d’Ivoire, Kouakou et al. [44] ascertain the absence of *S*. *gesnerioides* in cowpea fields. To the northwest, occurrences were only recorded in southeastern Mali and western Senegal. In the latter country, most data concern the groundnut basin, and the *Striga* infestation appears less severe, as compared to the center and east of West Africa [24].

### Bioclimatic envelopes of cowpea and cowpea-*Striga* infestation

**Fig 2:**
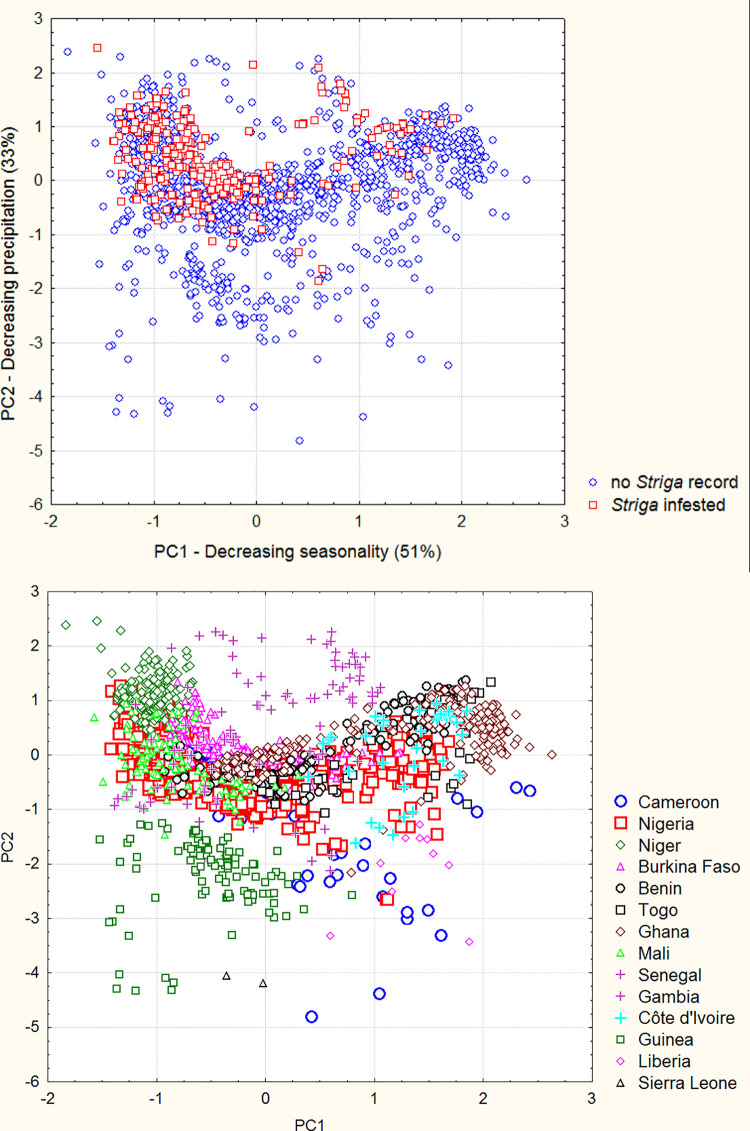
Principal component analysis (varimax normalized rotation) of cultivated cowpea ecoclimatic envelope. Dispersion of datapoints in the principal plane contrasting (**A**) reported infestations of the crop or (**B**) observations from distinct countries.

Table 1 and Fig 2 present the results of the PCA characterizing the ecoclimatic envelopes of cultivated cowpea. Bioclimatic variables 1 (annual mean temperature), 2 (mean diurnal range), 6 (minimal temperature of coldest month), 9 (mean temperature of driest quarter), 11 (mean temperature of coldest quarter), 14 (precipitation of driest month), 17 (precipitation of driest quarter) and 19 (precipitation of coldest quarter) were excluded from the analysis because of their poor specific contribution to the ecoclimatic model and/or because they can be deduced directly from other bioclimatic variables. The two principal components with an eigenvalue superior to 1 are related to (i) seasonality of temperatures and precipitation (explaining 51% of the total variance), and (ii) precipitation and temperature of the wettest month and quarter (33% of the total variance).

**Table 1:** Principal component analysis (varimax normalized rotation) on cultivated cowpea ecoclimatic envelope.

Bioclimatic variables	PC 1	PC 2
3- Isothermality	**0.92**	-0.08
4- Temperature seasonality	**-0.87**	0.38
5- Maximal temperature of warmest month	**-0.90**	0.25
7- Temperature annual range	**-0.94**	0.04
8- Mean temperature of wettest quarter	-0.31	**0.75**
10- Mean temperature of warmest quarter	**-0.76**	0.47
12- Annual precipitation	0.57	**-0.77**
13- Precipitation of wettest month	0.10	**-0.95**
15- Precipitation seasonality	**-0.84**	0.29
16- Precipitation of wettest quarter	0.13	**-0.97**
18- Precipitation of warmest quarter	**0.79**	-0.30
Explained variance	5.65	3.61
Proportion of total variance (%)	51	33

Factor loadings of most important bioclimatic variables onto the first two components (values higher than 0.70 in bold characters).

Cultivated cowpea occurrences clearly accumulate at high values of the second principal component, i.e., under semi-arid climates, characterized by a severe dry season of 7–10 months and only 1–2 months of rainfall in the north to 4–5 months in the south, with a high spatial, inter- and intra-annual variability (Fig 2A). This accumulation of datapoints in the upper part of the principal plane seems to draw a saddle-shaped aridity limit to the cowpea climatic envelope; only ca. 60 occurrences, mostly from Senegal (Fig 2B), lie above this limit.

The vast majority of *Striga* infestations are observed in this densest and driest part of the cowpea climate envelope, particularly under highly contrasted climates. Clearly, *S*. *gesnerioides* is more xerophytic than cowpea and its aggressiveness is favored by aridity. The identification of datapoints by country (Fig 2B) helps interpreting the geography of *S*. *gesnerioides* impact. Niger and northern Senegal are the places where cowpea is confronted to the most arid and contrasted climates, closely followed by Mali, Burkina Faso, northern Nigeria and northern Cameroon. Along the Dahomey gap, climates of Ghana, Togo and Benin present slightly less arid and less contrasted climates, and these countries are less severely exposed to *Striga* attacks. In contrast, cowpea cultivation areas from Guinea, Liberia, southern Cameroon and southern Senegal, in the lower half of the principal plane, i.e., under much less arid climates, do not appear to be threatened by *Striga* parasitism. The case of Côte d’Ivoire is particular, as its northeastern region presents climates similar to those of Benin, Togo and Ghana, but no cases of *Striga* parasitism on cowpea.

### *Striga gesnerioides* in West Africa: Hosts, geography and evolution of collections

In addition to the 275 records of cultivated cowpea parasitism, 91 cases were reported on other species, and 59 cases without host information were recovered, for a total of 425 occurrences of *S*. *gesnerioides* (Table 2). Thus, in the study area, *S*. *gesnerioides* parasitizes many dicotyledons, mostly wild members of Fabaceae (*Alysicarpus ovalifolius*, *Cassia mimosoides*, *Indigofera astragalina* and *Indigofera sp*., *Tephrosia elegans and T*. *pedicellata*, and *Zornia glochidiata*) and Convolvulaceae (*Ipomoea coptica*, *I*. *coscinosperma*, *I*. *eriocarpa*, *I*. *nil*, *I*. *pescaprae*, *I*. *pes-tigridis*, *I*. *pileata*, *I*. *vagans*, *Jacquemontia tamnifolia*, *Merremia pinnata* and *M*. *tridentata*). It has even been reported on an introduced legume, *Arachis repens*, in Nigeria and Togo [45]. More rarely, it has been found on species of the Primulaceae (*Anagalis pumila*). The single observation on cultivated tobacco (*Nicotiana tabacum*, Solanaceae) is not surprising, as *S*. *gesnerioides* is a major pest of this crop in Zimbabwe [21]. More surprising are the four cases on monocotyledons from genera *Andropogon* and *Pennisetum* (*P*. *pedicellatum*) in Mali and Benin. Once compiled from the available databases and literature, this host range appears wider than previously published lists [19–24, 44, 46, 47].

**Table 2:** Number of *S*. *gesnerioides* parasitism records per host and country in West Africa. There were no records for Guinea, Guinea Bissau, Liberia, and Sierra Leone.

Countries of collection												
Hosts	Niger	Nigeria	Cameroon	Benin	Togo	Burkina Faso	Ghana	Mali	Côte d’Ivoire	Gambia	Senegal	Total
***Fabaceae***												
***Vigna unguiculata***	51	46	12	28	7	78	13	18		1	21	**275**
***Alysicarpus ovalifolius***						1						**1**
***Arachis repens***		1			3							**4**
***Indigofera astragalina***											3	**3**
***Indigofera sp***.				2		1		4				**7**
***Tephrosia elegans***		1	1	7		1			8			**18**
***Tephrosia pedicellata***						2						**2**
***Zornia glochidiata***											1	**1**
**Other Fabaceae**			2					1				**3**
**Convolvulaceae**												
***Ipomoea coptica***											1	**1**
***Ipomoea coscinosperma***						1						**1**
***Ipomoea eriocarpa***						1			14			**15**
***Ipomoea nil***						1						**1**
***Ipomoea pescaprae***											1	**1**
***Ipomoea pes-tigridis***								1				**1**
***Ipomoea pileata***	1											**1**
***Ipomoea sp***.						1		1			2	**4**
***Ipomoea vagans***											4	**4**
***Jacquemontia tamnifolia***	2	1										**3**
***Merremia tridentata***											4	**4**
***Merremia pinnata***								1				**1**
**Caesalpiniaceae**												
***Cassia mimosoides***						1						**1**
**Commelinaceae**												
**Primulaceae**												
***Anagalis pumila***				1								**1**
**Solanaceae**												
***Nicotiana tabacum***	1											**1**
**Poaceae**												
***Andropogon amethystinus***				1								**1**
***Andropogon sp***.				1								**1**
***Pennisetum pedicellatum***								1				**1**
**Wild**		4		4				1				9
**Uncategorized**	2	1	2	30	4	4	3	8	1		4	59
**Total**	**57**	**54**	**17**	**74**	**14**	**92**	**16**	**36**	**23**	**1**	**41**	**425**

**Fig 3:**
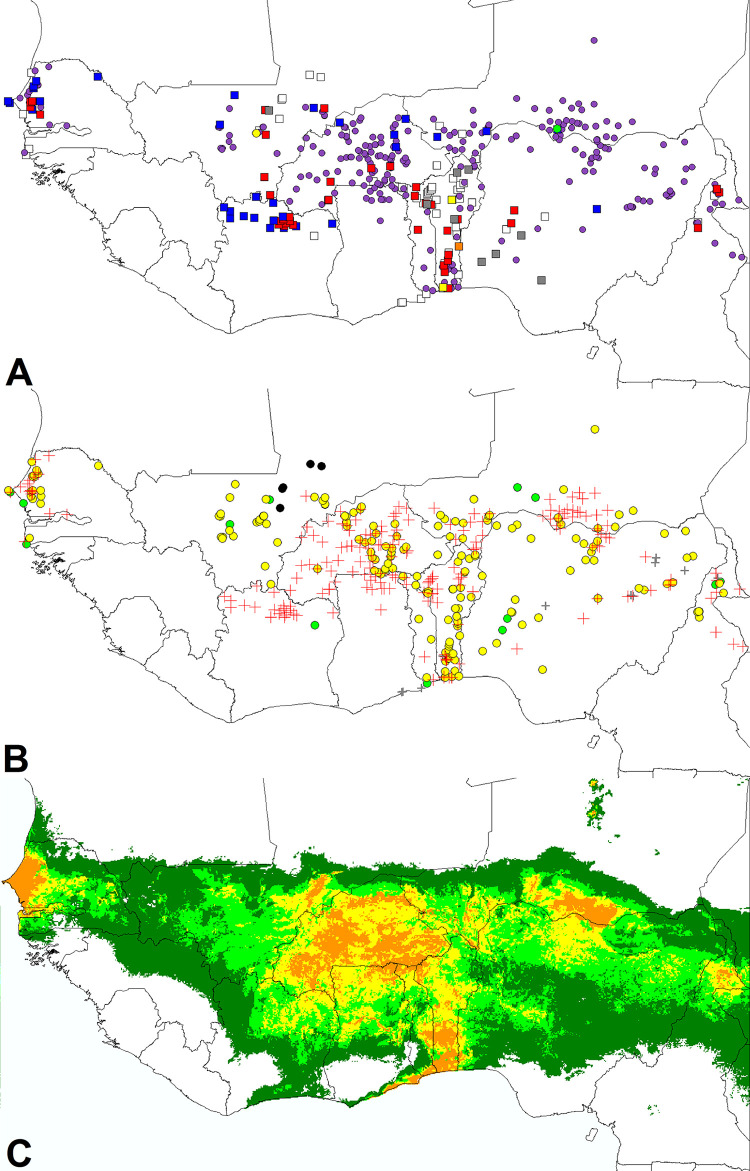
Distribution of *S*. *gesnerioides* records and derived distribution model. **A.** Occurrence records categorized by host species: cowpea (pink circles), wild Fabaceae (red squares), wild Convolvulaceae (blue squares), Poaceae (yellow squares, for *Andropogon*, and circle, for *Pennisetum*), *Anagalis pumila* and *Cyanotis lanata* (orange squares), indeterminate wild hosts (grey squares) and indeterminate hosts in indeterminate environment (white squares). **B.** Occurrence records categorized by decadal or bidecadal period of observation: 1899–1909 (black circles), 1960–1980 (green circles), 1980–2000 (yellow circles), 2000–2020 (red crosses), unknown date (grey crosses). **C.** Maxent ecoclimatic distribution model derived from these observations (color code as for Fig 1B).

Mapping the occurrences of *S*. *gesnerioides* (Fig 3A) first shows its quite general presence in the Sahelian and Sudano-Sahelian belts and its absence to the south, except in Benin and relatively close areas of Togo and Nigeria, in relation to the dry climate of the Dahomey gap. Second, no clear geographic differentiation can be detected between the distribution of *S*. *gesnerioides* hosted by cowpea and that of records on other hosts (wild plants in their great majority). However, again we find distinct trends in eastern and western parts of West Africa. To the east, in Niger, Nigeria, and Burkina Faso, observations on cowpea appear more widespread than on wild plants; only in Benin do the reports on wild plants cover a significant area of the country, comparing well with that observed on cowpea. To the west, the cases on cowpea and on other plants appear much better balanced, both in their geographic distribution and in their numbers. An extreme case is that of northern Côte d’Ivoire, where all observations concern wild plants. One likely reason for the higher prevalence of cowpea specific strains in eastern West Africa is that cowpea itself is more present there, which may cause a collecting bias in our host data. A second one is that *S*. *gesnerioides* has become much more common there because of this earlier extension and intensification of cowpea cultivation to the east. The latter explanation is consistent with the historical effect of monocropping and loss of soil fertility on the severity of the parasitism [16, 17, 20, 25, 30, 31]. Thence it is important to understand both the spatial and temporal dynamics of the *S*. *gesnerioides* problem aggravation. To address this question, we have categorized the observations by bi-decadal periods (Fig 3B).

Up to 1980, it seems that *S*. *gesnerioides* was quite neglected by botanists and agronomists. Indeed, the five earliest collections of our dataset, dating from 1899 to 1909, are all from Mali, and their host was not identified. The next 15 were collected in Cameroon, Niger, Nigeria, Togo, Côte d’Ivoire, Mali and Senegal, from 1960 to1980. The host was identified only in six cases, four on cowpea and two on wild plants (determined to family or genus). For the 1980–2000 bidecadal period, the number of records increases very significantly, with a total of 186, in the same seven countries mentioned above, plus Burkina Faso and Benin. The host was often recorded too, cultivated cowpea being most common: four out of five in northern Cameroon, 14 out of 18 in Niger, 22 out of 29 in Nigeria, 28 out of 32 in Burkina Faso, and 17 out of 26 in Mali. The picture was slightly different for Benin, where the numbers of cowpea (19) and wild hosts (12) were more balanced, thanks to a phytosociological inventory by a group from the Abomey-Calavi University. It was even more different for Togo, with only six cases, two of which on cowpea, and for Senegal, with 15 records on wild hosts versus five on cowpea. More globally, the sudden increase in record numbers was related to a series of systematic field surveys, themselves justified by the increasingly severe impact of *S*. *gesnerioides* parasitism on cowpea cultivation, such as the FAO study in northern Cameroon, Nigeria, Niger, Benin and Togo, [45, 48], that of Wade [24] in Senegal, and the extensive collections of herbarium materials by A. Raynal-Roques in Burkina Faso and Mali, from 1985 to 1991. Efforts to develop genetic resistance also provided information on the distribution of *S*. *gesnerioides* races [27].

From 2000 to present, collecting efforts have further increased, in Niger (Maradi and Zinder regions; [49, 50], Benin, northern Ghana (nine sites; [51], Burkina Faso (58 sites; [47], and in the groundnut basin of Senegal [52]. Our dataset includes 207 records (after validation and filtering), with 156 cases on cultivated cowpea, and 32 on other plants, mostly contributed from a survey in Côte d’Ivoire, where *S*. *gesnerioides* was observed only on wild hosts [44].

While the chronological dynamics of data accumulation clearly indicates that *S*. *gesnerioides* had become a very significant pest on cowpea around 1980, more precise information on the spatio-temporal sequence of this development is quite scarce and difficult to scan, because of the successive delays between the occurrence of the problem, the related research programs, and the publication of their results.

At the latest, the problem appeared in the late 1970s in the cowpea cultivation basin of Niger and northern Nigeria, as well as in southern Benin. Indeed, following a five-year survey in Niger, from 1981 to 1985, Adam [12] identified *S*. *gesnerioides* as one of the three major causes of the decreasing cowpea yield, in relation to its abundance in the central-eastern (Maradi and Zinder) and western (Niamey) regions of the country. In the proceedings of the Second International Workshop on *Striga*, held in 1981, Obilana [53] commented on the serious threat posed by *S*. *gesnerioides* in northern Nigeria, with very severe crop losses in 1980, and mentioned field screening for resistance or tolerance to this pest, in 1977, 1978, and 1979. According to the report of Parkinson [45] on his 1984–1985 survey, *S*. *gesnerioides* attacks on cowpea were limited to the Sudano-Sahelian region of Nigeria, although it was observed much further south on other hosts. He further mentioned cowpea as the host of *S*. *gesneroides* in southern Benin, farmers claiming “that most of the cowpea consumed in Benin used to be produced in Zou Province but in the last five years, this is no longer true because of *Striga* infestation.” Thus, *S*. *gesnerioides* infestation was very severe before 1980 in this region too.

Countries in the center and west of the studied region suffered attacks later: in the above-mentioned Second International Workshop on *Striga* of 1981, Reneaud [54] commented that in the Volta valleys (Burkina Faso), *S*. *gesnerioides* was still considered of minor importance, in a context of sporadic cultivation of cowpea on limited surfaces. For northern Ghana, Stoop et al. [55] did not even mention cowpea among the *Striga*-threatened crops, and Parkinson [45] found no incidence of infestation of cowpea in Togo in 1984–1985, despite the observation of *S*. *gesnerioides* parasiting *Arachis repens*.

This eastward delay in *S*. *gesnerioides* infestation was confirmed in 1990 by Cardwell and Lane [30], whose systematic survey revealed more general and severe attacks on cowpea in Niger, northern Nigeria and southern Benin, than in Burkina Faso and Mali. Of notice too is the observation of Hoffmann et al. [56] that cowpea infestations were still localized and limited to small areas in Mali in 1991. Even further west, in Senegal, the impact of *S*. *gesnerioides* was marginal in the 1970s [57]. It became increasingly significant after 1985, following the collapse of peanut production and the extension of cowpea cultivation [24, 52]. For the period 1993–1996, Wade [24] documented the presence of *S*. *gesnerioides* on wild plants and, increasingly, on cultivated cowpea in most of the peanut production basin (14% of cowpea fields infested), and more particularly in its central and northern portions, developing important populations in departments where cowpea was more prominent (50% of infested fields in certain villages). Furthermore, Wade [24] demonstrated that the parasite was able to shift successfully between wild hosts and cowpea, in both directions, and predicted that it would soon become a considerable threat for the crop. The studies of Wade [24] and Tonessia et al. [52] constitute the clearest field demonstration of the relationship between the expansion of cowpea cultivation (from 12,000 ha in 1970 to 90,000 ha in 2001) and its parasitism by *S*. *gesnerioides*, further aggravated by the introduction of susceptible cowpea cultivars and the development of virulence against the most common cultivars.

This spatiotemporal analysis of the literature leads us to use a similar narrative to explain the spread of severe *S*. *gesnerioides* parasitism on cowpea at the scale of the whole region. Before the rise of infestations, both the crop and the parasite had the same distribution as today in West Africa, and the general intensification of cowpea production has led to the current situation, first in two early cowpea basins of (i) northern Cameroon, Niger, Northern Nigeria and (ii) southern Benin, and later westward to Togo, Ghana, Burkina Faso, Mali and Senegal. Contrary to the vision of a wide-scale diffusion of new and aggressive forms of the parasite, following the spread of their host, the multilocal vision derived from Wade’s interpretation implies that the distribution of *S*. *gesnerioides* was already in equilibrium well before the expansion and intensification of cowpea cultivation, which can be verified by ecoclimatic modeling and a comparative study of the bioclimatic envelopes on wild hosts and cowpea.

### Global distribution model of *Striga gesnerioides*

Given the apparent absence of geographical differentiation among *S*. *gesnerioides* strains affecting different hosts, Maxent was run on the whole occurrence dataset, producing the distribution model presented at Fig 3C. In the resulting ecoclimatic model, the distribution of suitable areas fits quite well to the data of *S*. *gesnerioides* parasitism. Other areas are not favorable to the parasite, so that the comparison with the cowpea model (Fig 1) indicates that wide cowpea cultivation areas, in Guinea, southern Côte d’Ivoire, southern Ghana, and southeastern Nigeria, are not significantly threatened by *S*. *gesnerioides*. This observation is clearly consistent with the results of our first PCA (Fig 2).

Our *S*. *gesnerioides* model is much more restrictive than the one obtained by Mohamed et al. [22]. Indeed, the latter differs in predicting (i) the presence of *S*. *gesnerioides* slightly more to the north; (ii) its absence only in Liberia and Sierra Leone, and not in the adjacent territories of Guinea and Côte d’Ivoire; (iii) its presence in central Cameroon, in many dry and humid forest regions, and even in the Congo basin (which is clearly contradicted by the extrapolation of our model).

### Bioclimatic envelope of *Striga gesnerioides*

**Fig 4:**
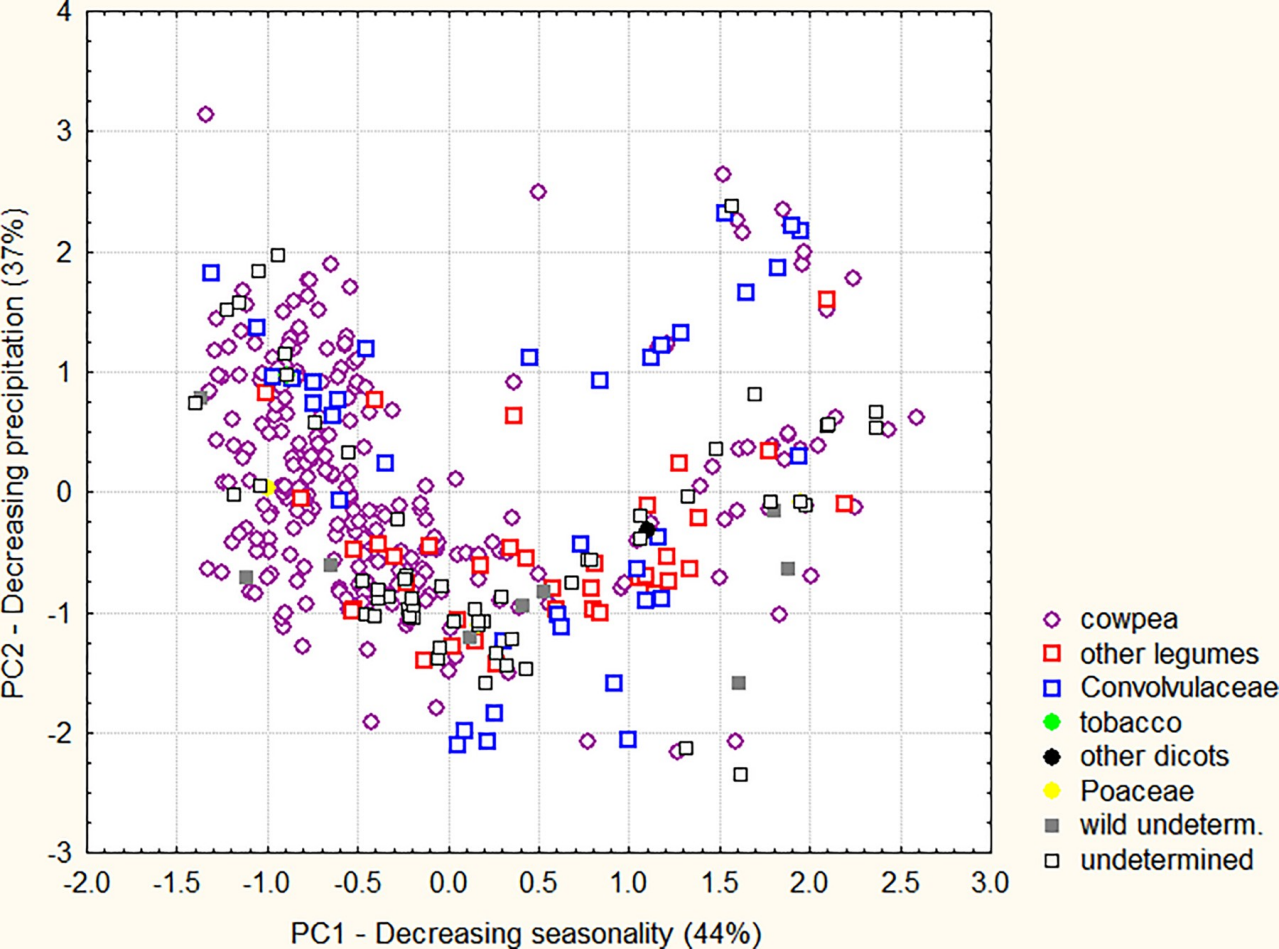
Principal component analysis (varimax normalized rotation) of *S*. *gesnerioides* bioclimatic envelope. Dispersion of datapoints in the principal plane, according to host species.

The *S*. *gesnerioides* Maxent model is based on the same bioclimatic variables that most contributed to the cultivated cowpea model. In the PCA, this remarkable similarity in the bioclimatic determinants of the distribution of the crop and its parasite extends to the orientation of the two factors with an eigenvalue superior to 1, which are related to (i) seasonality of temperatures and precipitation (44% of the total variance), and (ii) precipitation and temperature of the wettest month and quarter (36% of the total variance) (Table 3). Furthermore, the distribution of *S*. *gesnerioides* occurrences in the principal plane (Fig 4) is clearly reminiscent of that of cultivated cowpea infestations (upper part of the principal plane presented in Fig 2), with a saddle-shaped concentration of points, and a few Senegalese occurrences above. While Fig 2 shows that the parasite has not adapted to the whole cultivated cowpea niche, Fig 4 shows no divergences between the distribution of cases on cowpea and those on wild hosts of any botanical family in the general climatic envelope of the parasite, pointing also to the absence of climatic specialization among *S*. *gesnerioides* strains affecting distinct hosts.

**Table 3:** Principal component analysis (varimax normalized rotation) on *S*. *gesnerioides* bioclimatic envelope.

Bioclimatic variables	PC 1	PC 2
**3- Isothermality**	**0. 89**	-0.08
**4- Temperature seasonality**	**-0.82**	0. 47
**5- Maximal temperature of warmest month**	**-0.91**	0. 23
**7- Temperature annual range**	**-0.88**	0. 30
**8- Mean temperature of wettest quarter**	-0.18	**0. 85**
**10- Mean temperature of warmest quarter**	**-0.82**	0. 30
**12- Annual precipitation**	0. 41	**-0.88**
**13- Precipitation of wettest month**	0. 09	**-0.89**
**15- Precipitation seasonality**	-0.47	0. 63
**16- Precipitation of wettest quarter**	0.15	**-0.94**
**18- Precipitation of warmest quarter**	**0. 77**	-0.09
**Explained variance**	4.79	4.03
**Proportion of total variance (%)**	44	37

Factor loadings of most important bioclimatic variables to the first two components (values higher than 0.70 in bold characters).

Thus, our analyses of both the geographic and ecoclimatic distributions of *S*. *gesnerioides* contradict Mohamed’s hypothesis that the wide distributions of *Striga* species is linked to their diversity of hosts [18, 22]. Instead, it seems that the distribution of *Striga* parasitism on cowpea corresponds to the simple intersection of geographic and climatic distributions of the crop and the parasite. To test this further, we have developed a *S*. *gesnerioides* distribution model only from infestations on cowpea, and compared it with the distribution model obtained from the superposition of the cultivated cowpea model (Fig 1B) and the general *S*. *gesnerioides* model (Fig 3C).

### Modeling the distribution of *Striga gesnerioides* infestations on cultivated cowpea

**Fig 5:**
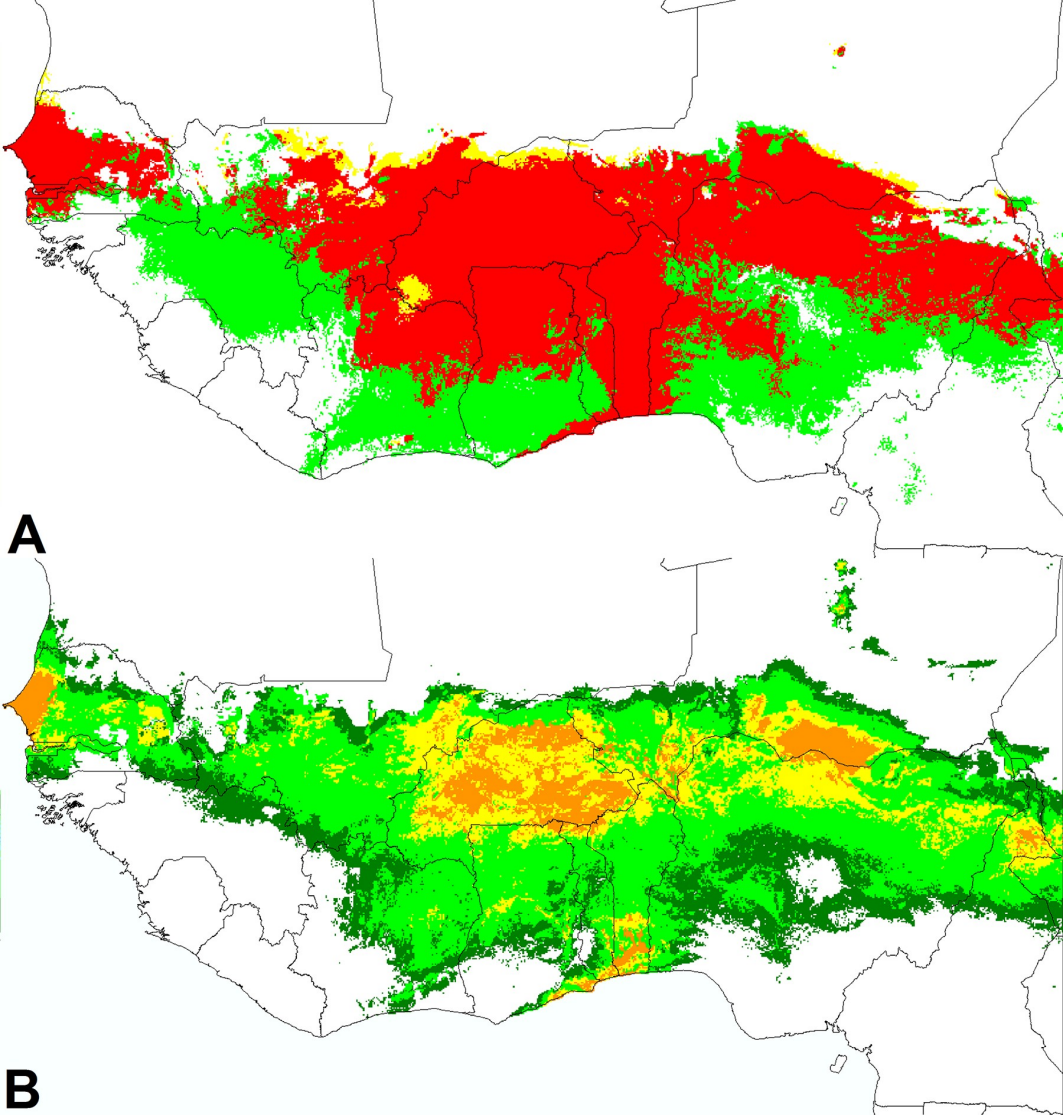
Models of distribution for infestation of cultivated cowpea by *S*. *gesnerioides*. **A.** Superposition of Maxent models for the crop (as presented in Fig 1B) and its parasite (as presented in Fig 3C); green areas: favorable only for cowpea cultivation; yellow areas: favorable only for *S*. *gesnerioides*; red areas: favorable for both species. **B.** Distribution model derived directly from observations of cultivated cowpea infestations (color code as for Fig 1B).

Fig 5 presents the superposition of the cultivated cowpea model and the global *S*. *gesnerioides* model (all hosts included; Fig 5A) and the model derived directly from the 275 observations of cultivated cowpea infested by *S*. *gesnerioides* (Fig 5B). On Fig 5A, the red areas represent regions where the cowpea model and the global *S*. *gesnerioides* model predict the presence of both the crop and the parasite; yellow areas represent rare regions where the parasite is present under climates that are not suitable or marginally suitable for the crop; green areas represent regions that are climatically suitable to the crop, but not suitable or marginally suitable to the parasite. The good overlap of the “superposition red model” and the cowpea infestation model confirms that the distribution of *S*. *gesnerioides* depends essentially on its own ecophysiological requirements, not on the ranges of its hosts. Remarkably, the two compared approaches converge for the particular case of northeastern Côte d’Ivoire, where both the crop and the parasite are effectively present, although no cowpea-specific strain of *S*. *gesnerioides* has been reported yet.

## Conclusion

Our analyses on the bioclimatic envelope and geographic range of *S*. *gesnerioides* contradict the hypothesis of Mohamed et al. [22], showing that they do not correspond to a mosaic of niches composed of those of its hosts. In fact, each of *S*. *gesnerioides* hosts has its own distribution, and the distributions of particular host-parasite combinations is determined only by the overlap of their respective niches and the evolution of local strains in response to the prevalence of the host. Not only does the distribution of *S*. *gesnerioides* appear to be in equilibrium, but also the spatial and temporal distribution of observations on wild plants clearly indicates that this was already the case before it became a serious cowpea pest. Therefore, an epidemic diffusion of *S*. *gesnerioides* attacks on cowpea would imply (i) a very strong specificity of the host-parasite relationship, as was suggested by the morphological differentiation of cowpea-specific strains of the parasite [18], and (ii) an efficient seed propagation. In this view, a major factor aggravating the severity of the attacks, in relation to the extension and intensification of cowpea cropping, is the long-term seed accumulation in the soil [58].

However, the observations and experiments of Wade [24] show that the solidity of the host-parasite specificity has been overestimated, and his predictions about the risk of more strains from the wild shifting to cowpea have been verified in the peanut basin of Senegal [52]. The process described there is the best model to interpret the spatiotemporal dynamics of the spread of infestations on cowpea across the whole region of our study. Thence, the extension and intensification of cowpea cultivation does not only appear to be an aggravating factor in terms of severity; in fact, it is the main driver of the evolution of pre-existing *S*. *gesnerioides* populations, in a wide multilocal process. In this model, the “invasive” species (in ecological terms) is not *S*. *gesnerioides* but *Vigna unguiculata*. Its overdominance in the agricultural landscapes and the consequent reduction of biodiversity induce the evolution of pre-existing local *Striga* strains.

A multilocal model is consistent with the doubts of Berner et al. [25] on the real contribution of long distance migration mechanisms in explaining the severity of the problem. Indeed, fast long-distance diffusion mechanisms cannot account for the observed parallelism between the time geography of the parasite expansion and that of the crop intensification. If fast diffusion prevailed, it would be more dependent on the situation in the source region, distance, and diffusion parameters than on the situation of the newly infested region. In the multilocal model, the parasite prolificity and mobility plays at a much smaller scale. This is also true for diffusion through contaminated cowpea seed because of social constraints on seed movements [59, 60]. The progressive diffusion of locally emerging cowpea-specific strains is also consistent with the most recent genetic analyses: “SSR analysis indicates that *Striga* populations are highly differentiated and genetic relatedness generally corresponds with geographic proximity rather than their host compatibility” [61].

The same causes producing the same effects, *S*. *gesnerioides* has become a constraint for the cultivation of sweet potato in Eastern Africa [20], and tobacco in Southern Africa [21]. In *S*. *hermonthica* too, the distribution of the parasite prevails on that of the potential hosts and host shifting takes place within it. Thus, Bellis et al. [36] obtained the same distribution pattern with 1050 observations on different hosts or with a subsample of 262 observations on sorghum alone. And impressive evolutions in *S*. *hermonthica* virulence have been observed, with rapid host changes in response to the introduction and expansion of new crop species [28, 62].

As argued by Botanga and Timko [26], the same host-driven selection plays a key role in the diversification of the parasite on a same host species, with the emergence of new races and virulence, breaking down varietal resistances. Thus, in the same way as the invasion of an agricultural landscape by the monoculture of cowpea and the suppression of fallows leads to a jump of the parasite from the spontaneous compartment to the cultivated one, the lack of varietal diversity induces the emergence of new virulences.

The *Striga* problem is one of evolutionary genetics and the response must be treated within this framework, taking full account of the agro-ecological dimension of the phenomenon (crop diversification, reintroduction of fallows, etc). For the breeder, it is particularly important to understand the evolutionary relationship between the diversification of *S*. *gesnerioides* across host species and that of geographical diversity of virulence across cultivars [26, 60]. Indeed, a better understanding of these evolutionary processes is crucial for improving and managing genetic resistance in cowpea cultivars/landraces to confront the increasing diversity of virulent *S*.*gesnerioides* strains.

## Supporting information

S1 Table Statistics on data collation (S1A), GBIF data cleaning (S1B) and final data selection (S1C). (DOCX)
