# Supplementary material for: The witchweed Striga gesnerioides and the cultivated cowpea: A geographical and historical analysis of their West African distribution points to the prevalence of agro-ecological factors and the parasite’s multilocal evolution potential
Source: PLoS One. 2021 Aug 4;16(8):e0254803. doi: 10.1371/journal.pone.0254803 (PMC8336835; doi:10.1371/journal.pone.0254803)
Supplement: S1 Table — (DOCX) [file pone.0254803.s001.docx]

**S1 Table**

**S1A Table**. *Vigna Unguiculata* (L.) Walp and *Striga gesnerioides* (Wild.) Vatke initial occurrences data sources used for geographical distribution of both cultivated cowpea and *Striga gesnerioides* in West Africa.

|  | Number of data per plant and acquisition period | | |
| --- | --- | --- | --- |
| Occurrences data sources | *Vigna Unguiculata*  (L.) Walp | *Striga gesnerioides (*Willd.) Vatke | Acquisition period |
| Field works within the framework of the CowpeaSquare project in Niger | 443 | 443 | September and october 2016, 2017 and 2018 |
| Field works within the framework of the CoEx projet | 144 |  | 29/01/2020 |
| Field works within the framework of the projet “Gestion durable de la biodiversité du niébé (Vigna unguiculata (L.) Walp) et espèces apparentées sauvages au Sénégal” | 37 |  | 04/06/2020 |
| - GBIF databases : (<https://www.gbif.org/occurrence/download/0292529-200613084148143>) for Vigna Unguiculata (L.) Walp ;   (<https://www.gbif.org/occurrence/download/0292543-200613084148143>) for Striga gesnerioides (Willd.) Vatke | 27153 | 719 | 04/03/2019 (for *Vigna Unguiculata*  (L.) Walp), and 29/08/2019 (for *Striga gesnerioides (*Willd.) Vatke) |
| MNHN (Muséum National d'Histoire Naturelle) : <https://science.mnhn.fr/all/list?scientificName=Striga+Gesnerioides> |  | 242 | 19/10/2019 |
| Herbier National du Bénin :( <http://publish.plantnet-project.org/project/herbierbenin> ) |  | 12 | 21/12/2019 |
| [1] | 5 | 18 |  |
| [2] | 1 | 1 |  |
| [3] | 1 | 1 |  |
| [4] | 8 | 8 |  |
| [5] | 48 | 54 |  |
| [6] | 2 | 33 |  |
| [7] | 1 | 1 |  |
| [8] | 22 | 2 |  |
| [9] | 29 | 7 |  |
| [10] |  | 22 |  |
| [11] | 8 | 8 |  |
| [12] | 38 | 38 |  |
| [13] | 8 | 8 |  |
| [14] | 1 | 2 |  |
| [15] | 14 | 14 |  |
| [16] | 5 | 5 |  |
| [17] | 1 |  |  |
| [18] | 4 | 1 |  |
| [19] | 19 | 19 |  |
| **Total** | **27992** | **1658** |  |

**References**

1. Wade M. *Striga gesnerioides* (Willd.) Vatke parasite du niébé dans le bassin arachidier du Sénégal: situation actuelle et perspectives de lutte : synthèse des travaux menés sur *Striga gesnerioides* de 1987 à 1998. Bambey: Centre National de Recherches Agronomiques. Available from: http://intranet.isra.sn/aurifere/opac_css/docnum/CN0103022.pdf

2. Kondi Y, Mawuli A, Agnassim B, Yentchabre P, Koffi T. Evaluation de quatre cultivars de niébé (*Vigna unguiculata* (L.) walp.) pour leur résistance au déficit hydrique et à l’adventice parasite, *Striga gesnerioides* (Willd.) Vatke au Togo. ESJ. 2018;14(6):215–228.Available from: https://eujournal.org/index.php/esj/article/view/10527/10060

3. Obilana AT. *Striga* studies and control in Nigeria. In Ramaiah KV, Vasudeva Rao MJ, editors: Proceedings of the Second International Workshop on *Striga*, 5-8 October 1981, Ouagadougou, Upper Volta. Patancheru (India): ICRISAT; 1983. pp. 87–98. Available from: http://oar.icrisat.org/784/1/RA_00047.pdf

4. Tonessia C, Wade M, Cissé N, Séverin A. Characterization of *Striga gesnerioides* from Senegal: reaction of various cowpeas (*Vigna unguiculata* (L.) Walp.) to *Striga gesnerioides* strains from Senegal. J. Appl. Biosci. 2009;24:1462–1476.

5. Sawadogo P, Batieno TBJ, Dieni Z, Sawadogo N, Ouedraogo TJ, Sawadogo M. Geographical distribution and alternate hosts of *Striga gesnerioides* (Willd.) Vatke in Burkina Faso. J. Appl. Biosci. 2020;145:14955–14964.

6. Parkinson VO. A survey of infestation of crops by *Striga* spp in Benin, Nigeria and Togo. Proc N S Inst Sci. 1989; 39: 1–9. Available from: https://DalSpace.library.dal.ca//handle/10222/34634

7. Kamara AY, Chikoye D, Ekeleme F, Omoigui LO, Dugje IY. Field performance of improved cowpea varieties under conditions of natural infestation by the parasitic weed *Striga gesnerioides*. Int J Pest Manag. 2008;54(3):189–195. Available from: https://doi.org/10.1080/09670870801930260

8. Abdourahmane M, Ibrahim B, Rabe M, Saïdou A-A, Amadou L, Tamo M. Étude diagnostique des principales contraintes de la culture du niébé (*Vigna unguiculata* (L.) Walp) dans les régions de Maradi et Zinder au Niger. Afrique Science. 2020;32–43.

9. Rabe MM, Baoua I, Adeoti R, Sitou L, Amadou L, Pittendrigh B, et al. Les déterminants socioéconomiques de l’adoption des technologies améliorées de production du niébé diffusées par les champs écoles paysans dans les régions de Maradi et Zinder au Niger. Int. J. Biol. Chem. Sci. 2017;11(2):744-756. Available from : https://doi.org/10.4314/ijbcs.v11i2.17

10. Kouakou CK, Akanvou L, Bi IAZ, Akanvou R, N’da HA. Distribution des espèces de *Striga* et infestation des cultures céréalières dans le Nord de la Côte d’Ivoire. Cahiers Agricultures. 2015; 24: 37-46. Available from: https://doi.org/10.1684/agr.2015.0734

11. Mohamed KI, Musselman LJ, Riches CR. The genus *Striga* (Scrophulariaceae) in Africa. Ann Missouri Bot Gard. 2001; 88(1): 60-103. Available from: https://doi.org/10.2307/2666132

12. Ohlson EW, Timko MP. Race structure of cowpea witchweed (*Striga gesnerioides*) in West Africa and its implications for *Striga* resistance breeding of cowpea. Weed Sci. 2020; 68: 125–133. Available from: https://doi.org/10.1017/wsc.2020.3

13. Haruna P, Asare AT, Asare-Bediako E, Kusi F. Farmers and agricultural extension officers perception of *Striga gesnerioides* (Willd.) Vatke parasitism on cowpea in the Upper East Region of Ghana. Adv Agric. 2018; 2018:1–11. Available from: https://doi.org/10.1155/2018/7319204

14. Touré M, Olivier A, Ntare BR, Lane JA, St-Pierre C-A. Inheritance of resistance to *Striga gesnerioides* biotypes from Mali and Niger in cowpea (*Vigna unguiculata* (L.)Walp.). Euphytica. 1997;94:273–278. Available from: https://doi.org/10.1023/A:1002927705470

15. Botanga CJ, Timko MP. Phenetic relationships among different races of *Striga gesnerioides* (Willd.) Vatke from West Africa. Genome. 2006;49(11):1351–1365. Available from: https://doi.org/10.1139/g06-086

16. Adam T. Diseases affecting cowpea (*Vigna unguiculata* (L.) Walpers) in Niger. Ambio. 1990;19(8):358–360. Available from: https://www.jstor.org/stable/4313739

17. Kanteh SM, Norman JE, Kassoh AF. Effect of Saim weed (*Chromolaena odorata*) Leaf Mulch Rates on agronomic traits and yield of cowpea in Sierra Leone. In Njala University, Sierra Leone; 2014 [cited 2019 Dec 11]. Available from: https://www.researchgate.net/publication/270510937_Effect_of_Saim_weed_Chromolaena_odorata_Leaf_Mulch_Rates_on_Agronomic_Traits_and_Yield_of_Cowpea_in_Sierra_Leone

18. Seyni Bodo B, Malam Issa O, Tidjani Adamou D, Ambouta KJ-. M, Marin B, Ponthieu M, et al. Connaissance locale de la variabilité de surface du sol et des contraintes associées pour la production du niébé en zone sahélienne du Niger. EGS. 2019;26(1):65–79.

19. Lane JA, Moore THM, Child DV, Cardwell KF. Characterization of virulence and geographic distribution of *Striga gesnerioides* on cowpea in West Africa. Plant Dis. 1996;80(3):299–301.

**S1B Table.** Number of datalines removed from GBIF downloaded data (GBIF records).

|  | *Vigna Unguiculata* (L.) Walp | | *Striga gesnerioides (*Willd.) Vatke | |
| --- | --- | --- | --- | --- |
| Cleaning pipeline process used | Number | % | Number | % |
| Records with missing latitude, longitude or locality information | 9708 | 37.28 | 231 | 36.21 |
| Rounded coordinates (that cannot be safely improved) | 348 | 1.34 | 55 | 8.62 |
| Records with duplicate coordinates | 3589 | 13.78 | 118 | 18.50 |
| Records with coordinates equal to zero | 5735 | 22.03 | 121 | 18.97 |
| Records with duplicate localities/cities | 5174 | 19.87 | 113 | 17.71 |
| Records from market places | 1300 | 4.99 | 0 | 0 |
| Records related to wild cowpea | 184 | 0.71 |  |  |
| Total | 26038 | 100 | 638 | 100 |

**S1C Table.** Number of final occurrence data selected for geographical distribution of both cultivated cowpea [*Vigna unguiculata* (L.) Walp] and *Striga gesnerioides* in West Africa after cleaning and filtering.

| *Vigna Unguiculata* (L.) Walp | | | | | |
| --- | --- | --- | --- | --- | --- |
|  |  | Removed data | | Selected data | |
| Occurrence data sources | Original data | Number | % | Number | % |
| Field studies | 624 | 194 | 31.09 | 430 | 68.91 |
| GBIF databases | 27153 | 26038 | 95.89 | 1115 | 4.11 |
| Relevant literature | 215 | 13 | 6.05 | 202 | 93.95 |
| Total | 27992 | 26245 | 93.76 | 1747 | 6.24 |
| *Striga gesnerioides (*Willd.) Vatke | | | | | |
|  |  | Removed data | | Selected data | |
| Occurrence data sources | Original data | Number | % | Number | % |
| Field studies | 443 | 423 | 95.49 | 20 | 4.51 |
| GBIF databases | 719 | 638 | 88.73 | 81 | 11.27 |
| collections of MNHN (Paris) and National Herbarium of Benin | 254 | 167 | 65.75 | 87 | 34.25 |
| Relevant literature | 242 | 5 | 2.07 | 237 | 97.93 |
| Total | 1658 | 1233 | 74.37 | 425 | 25.63 |
